# Spinal stability analysis of lumbar interbody fusion according to pelvic type and cage angle based on simplified spinal model with various pelvic indices

**DOI:** 10.3389/fbioe.2022.1002276

**Published:** 2022-10-07

**Authors:** Cheol-Jeong Kim, Seung Min Son, Sung Hoon Choi, Dongman Ryu, Chiseung Lee

**Affiliations:** ^1^ Department of Biomedical Engineering, Graduate School, Pusan National University, Busan, South Korea; ^2^ Department of Orthopaedic Surgery, Pusan National University Yangsan Hospital, Yangsan, South Korea; ^3^ Department of Orthopaedic Surgery, Hanyang University College of Medicine, Seoul, South Korea; ^4^ Medical Research Institute, Pusan National University, Busan, South Korea; ^5^ Department of Convergence Medicine and Biomedical Engineering, School of Medicine, Pusan National University, South Korea; ^6^ Biomedical Research Institute, Pusan National University Hospital, Busan, South Korea

**Keywords:** pelvis index, simplified model, lumbar flexibility, spine stability, finite element analysis, lumbar interbody fusion

## Abstract

Recently, the objectives of lumbar interbody fusion (LIF) have been extended to include the correction of broader/relative indications in addition to spinal fixation. Accordingly, LIF must be optimized for sagittal alignment while simultaneously achieving decompression. Therefore, a representative model classified into three pelvic types, i.e., neutral pelvis (NP), anterior pelvis (AP), and retroverted pelvis (RP), was selected according to the pelvic index, and LIF was performed on each representative model to analyze Lumbar lordosis (LL) and the corresponding equivalent stress. The finite element (FE) model was based on a sagittal 2D X-ray image. The calculation efficiency and convergence were improved by simplifying the modeling of the vertebral body in general and its posterior portion in particular. Based on the position of the pelvis, according to the pelvic shape, images of patients were classified into three types: AP, RP, and NP. Subsequently, representative images were selected for each type. The fixation device used in the fusion model was a pedicle screw and a spinal rod of a general type. PEEK was used as the cage material, and the cage shape was varied by using three different cage angles: 0°, 4°, and 8°. Spinal mobility: The pelvic type with the highest range of motion (ROM) for the spine was the NP type; the AP type had the highest LL. Under a combination load, the NP type exhibited the highest lumbar flexibility (LF), which was 2.46° lower on average compared to the case where a pure moment was applied. Equivalent stress on the spinal fixation device: The equivalent stress acting on the vertebrae was lowest when cage 0 was used for the NP and AP type. For the RP type, the lowest equivalent stress on the vertebrae was observed when cage 4 was used. Finally, for the L5 upper endplate, the stress did not vary significantly for a given type of cage. In conclusion, there was no significant difference in ROM according to cage angle, and the highest ROM, LL and LF were shown in the pelvic shape of NP type. However, when comparing the results with other pelvic types, it was not possible to confirm that LF is completely dependent on LL and ROM.

## Introduction

Currently several surgical techniques are available to stabilize the spine (de Kunder et al., 2018). The LIF technique, which uses a cage, is performed to achieve neural decompression, bony fusion, and restoration of lumbar lordosis (LL) (Choi et al., 2017; Radovanovic et al., 2017; Park et al., 2018). However, the overall alignment relationship between the LL prediction of spinal surgery and the position of the pelvis and balance of the spine has not been adequately studied. Furthermore, the latest finite element modeling (FEM) studies of cage-inserted LIF focus primarily on the analysis of cage shape, material, and location (Cappuccino et al., 2010; Kim et al., 2014; Kim et al., 2017; Robertson et al., 2018; Zhang et al., 2018).

Consideration of sagittal spinal alignment arose with the evolution of operative treatment in adolescent idiopathic scoliosis (AIS) in the late 1980s (Thomson and Renshaw, 1989). Since Legaye and Duval-Beaupère introduced pelvic incidence (PI) as a key parameter regulating sagittal spinal balance, sagittal balance and its correlation with the results of spine surgery have been widely studied. PI is considered a constant parameter with no significant change with age, while thoracic kyphosis (TK) increases and lumbar lordosis (LL) decreases with age (Asai et al., 2017; Pratali et al., 2018).

Sagittal spino-pelvic alignment describes spinal and pelvic orientation in the erect posture with radiographic parameters. The adult deformity classification describes spinal deformity two-dimensionally with coronal curve types and three sagittal modifiers (Schwab et al., 2012). A correlation has been found between the shape and orientation of the pelvis and the morphology of sagittal spinal curvatures in asymptomatic persons (Berthonnaud et al., 2005). Decreased LL has been shown to have a strong correlation with low back pain (Chun et al., 2017).

In the study of Hatakka (Hatakka et al., 2021), in conclusion, the quality of the evidence on the effect of decompressive surgery for spinal-pelvic alignment was low, and there was substantial heterogeneity of the study design among the studies included. In addition, few studies have been conducted on the correlation of sagittal parameters according to LIF for each pelvic type. Accordingly, this study was conducted to analyze the correlation of the sagittal alignment according to the LIF using a numerical method rather than the statistics of clinical study results.

At first, in this study, a representative model of the version of the pelvis was selected based on the sagittal plane parameters determined from sagittal X-ray images. The position of the pelvis is adjusted by pelvic compensation with the hip joint as the axis, which regulates sagittal balance with respect to the line of gravity. Pelvic position (version) is largely divided into anteversion, neutral (equilibrium), and retroversion (Laouissat et al., 2018). Using classification according to the pelvic shape of choi, representative images of the anterior pelvis (AP) retroverted pelvis (RP), and neutral pelvis (NP) were selected (Choi et al., 2020).

To achieve posterior decompression, as shown in Figure 2, L4-L5 were fixed with a pedicle screw and rod, and the disc was removed to insert a bullet-shaped cage. Subsequently, surgery was simulated in which the cage was wrapped with a local bone graft and inserted as anteriorly as possible into the disc space before applying compression using posterior instrumentation (Salem et al., 2018). Finally, the range of motion (ROM), LL, and lumbar flexibility (LF) were measured to confirm spinal mobility resulting from the chiropractic operation in which the cage was inserted, and equivalent stresses in each area were calculated. The purpose of the experiment was to analyze lumbar flexibility and fixation stability according to pelvic type by measuring post-surgical ROM and equivalent stress. For this purpose, various sagittal plane alignment surgeries were virtually performed according to the above simulation results.

## Materials and methods

### Human lumbar spine FE model

**FIGURE 1:**
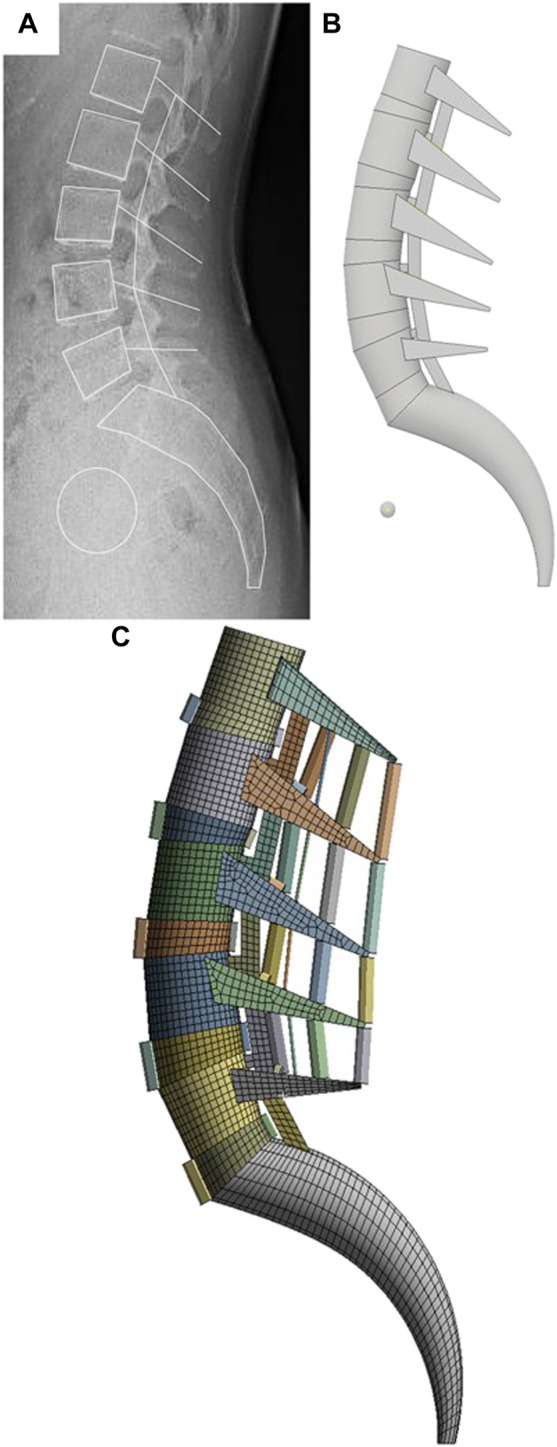
FE modeling generation sequence in this study. **(A)** Sagittal outline sketch of the vertebrae on the X-ray image. **(B)** Simplified 3D modeling based on sketch lines. **(C)** FE model generation using FEA program.

In general, the source of the finite element model is a 3D CT image; however, in this study, a 2D sagittal X-ray image was selected as the source to simplify the modeling based on the generated line projected on the sagittal plane. As shown in Figure 1A, the contours of the spine were drawn using the Autocad (Version 2019; Autodesk, Mill Valley, CA, United States) program to generate the 2D x-ray image as a 3D model. The generated spine contours are consistent with the Pelvic indicators in Table 1. and the posterior part of the vertebral body was simplified with the maximum length observed in the sagittal plane and the Facet joint connection line as the centerline. The IVD was extruded in the same shape on the upper and lower surfaces of the vertebral body. The simplification method of the lumbar spine 3D model applied in this study will contribute to modeling the entire spine in follow-up study.

**FIGURE 2:**
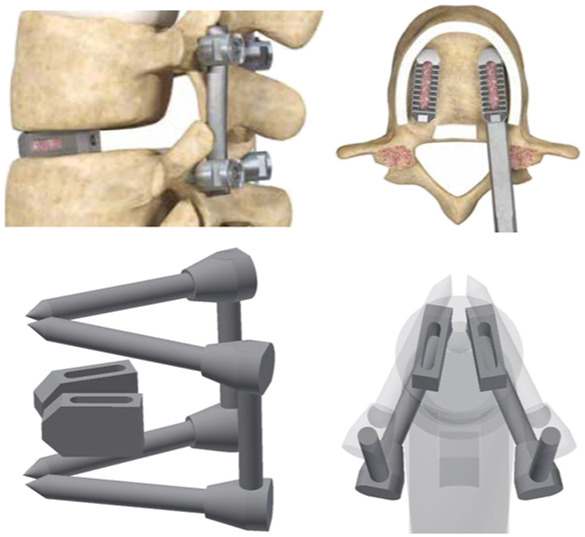
3D modeling that simulated LIF’s surgical method.

**TABLE 1:** Pelvic indicators for each representative model.

Pelvis Type	Lumbar lordosis (LL)	Pelvic incidence (PI)	Sacral slope (SS)	Pelvic tilt (PT)	SS/PI
Neutral pelvis (NP)	−56.4	54.9	43.0	11.9	0.783
Anteverted pelvis (AP)	−63.5	56.2	50.7	5.5	0.902
Retroverted pelvis (RP)	−43.1	59.1	33.5	25.6	0.567

**FIGURE 3:**
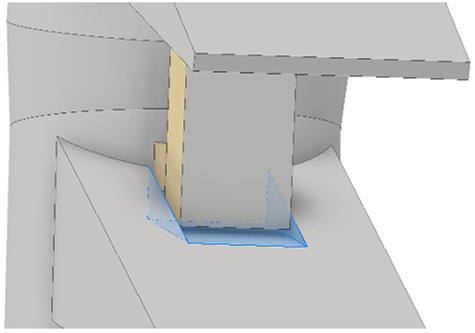
Simplify the facet joint of the vertebral arch.

In terms of modeling, the posterior part of the spine is the most difficult to simplify owing to its complex shape with several ligaments connecting the upper and lower parts. However, a simplified method for the posterior part of the spine based on the study by Goertz (Goertz et al., 2020), was applied, as shown in Figure 3. A rectangular post was created between the upper and lower parts of the posterior part of the spine to limit excessive ROM during movements such as posterior extension. A wedge-shaped slot was created in the portion in contact with the rectangular pole to limit excessive ROM during movements such as axial rotation. A certain gap was set between the column and slot to limit ROM when the two components come into contact owing to the applied motion. In the case of the facet joint, the shape differs from the full 3D model type; however, the function remains unchanged; therefore, soft frictionless conditions were applied as constraints to the model, and the initial interval was implemented as 0.5 mm (Rohlmann et al., 2009).

The vertebral segmentation range of the finite element model was L1–S1, as shown in Figure 1. The vertebral bone comprises cortical, cancellous, and posterior bones. The vertebra comprises the vertebral body and vertebral arch. The vertebral body is coated with the cortical bone, which is approximately 1 mm thick; the interior is filled with cancellous bone.

In the past Silva’s study (Silva et al., 1994), the cortical bone thickness measurement using CT was slightly lower than about 1 mm, and in Treece’s study (Treece and Gee, 2015), the femoral cortical thickness measurement using clinical CT was about 1–3 mm. Fazzalari measured the cortical bone thickness of the vertebral body at a maximum of approximately 1 mm (Fazzalari et al., 2006). Therefore, a 1 mm-thickness mesh with cortical bone properties was applied to the surface of the vertebral body in the FE model of this study, and a mesh with cancellous bone properties was used for the interior of the vertebral body. In addition, the material properties of posterior bone were applied to the vertebral arch.

Kim reported that the angle of correction of lumbar lordosis varies according to the angle of the cage inserted in spinal surgery (Kim et al., 2014). This study was conducted to analyze the changes in the sagittal parameters of the pelvic shape as well as the cage angle by developing Kim’s study. Therefore, the cage used in LIF of this study was simulated by the double cage structure applied in the clinical study of kim.

But, recently most of the cage surgery methods used for lumbar correction and fixation are LLIF (Lateral lumbar interbody fusion), and Oikawa’s study (Oikawa et al., 2022) also reported that LLIF has higher cage stability than PLIF (Posterior lumbar interbody fusion). Although as in Qin’s study (Qin et al., 2022) LLIF has stability issues according to the cage position, the most commonly used cage surgery method is LLIF. Therefore, in followe-up study, it is necessary to analyze the effect on the overall spinal sagittal plane by adding various surgical methods including LLIF to the spinal fixation surgery method and extending the interpretation range to the thoracic spine.

Anisotropic material properties were used for both the cortical and cancellous bones, as listed in Table 2, to calculate the tensile and compressive stresses. Here, the axial direction of the anisotropic material property (x-axis) is the forward/backward (flexion/extension) direction from the center of the FE model, and the Y-axis is the left/right lateral direction (lateral bending). The z-axis is the central axis of axial rotation in the direction of gravity (Zhong et al., 2009).

**TABLE 2:** Material properties

Material		Element type (ANSYS)	Young’s modulus (MPa)	Poisson’s ratio	Cross-section area (mm^2^)	Reference
Vertebra	Cortical bone	8-node Structural Shell (SHELL281)	E_X_ = 11,300	U_XY_ = 0.484	-	Zhong et al. (2009)
			E_Y_ = 11,300	U_YZ_ = 0.203		
			E_Z_ = 22,000	U_YX_ = 0.203		
			G_X_ = 3800			
			G_Y_ = 5400			
			G_Z_ = 5400			
	Cancellous bone	10-node Solid Element (SOLID187)	E_X_ = 140	U_XY_ = 0.45	-	
			E_Y_ = 140	U_YZ_ = 0.315		
			E_Z_ = 200	U_YX_ = 0.315		
			G_X_ = 48.3			
			G_Y_ = 48.3			
			G_Z_ = 48.3			
	Posterior bone (including Slot and post)	20-node Solid Element (SOLID186)	3,500	0.25		Zhang et al. (2018)
Intervertebral Disk	Ground substance	20-node Solid Element (SOLID186)	Hyper-elastic	-	-	Dooris et al. (2001), Ayturk et al. (2010), Casaroli et al. (2017)
			Mooney–Rivlin			
			C_1_ = 0.3, C_2_ = −0.9			
	Nucleus pulposus	8-node Fluid Element (FLUID30)	1	0.499	-	
Screw	Ti6Al4V	20-node Solid Element (SOLID186)	110,000	0.3	-	Zhang et al. (2018)
Spinal rod						
Cage	PEEK	20-node Solid Element (SOLID186)			-	Kurtz and Devine, (2007)
Ligament	ALL	2-node Link Element (LINK180)	7.8(ε < 12%) 20(ε > 12%)	-	63.7	Goel et al. (1993), Chen et al. (2001), Chuang et al. (2013), Kim et al. (2015)
	PLL		10(ε < 11%) 20(ε > 11%)	-	20	
	LF		15(ε < 6.2%) 19.5(ε > 6.2%)	-	40	
	ITL		10(ε < 18%) 58.7(ε > 18%)	-	1.8	
	ISL		10(ε < 14%) 11.6(ε > 14%)	-	40	
	SSL		8(ε < 20%) 15(ε > 20%)	-	30	
	CL		7.5(ε < 25%) 33(ε > 25%)	-	30	
Facet joint to Posterior Contact condition	Soft contact, Frictionless, Initial: 0.5 mm					Rohlmann et al. (2009)

The IVD comprises the nucleus pulposus and annulus fibrosus, of which the nucleus pulposus is an incompressible fluid (Dooris et al., 2001; Ayturk et al., 2010). The IVD was subdivided into the nucleus pulposus (56%) and annulus fibrosus (44%) and a hyper-elastic Mooney–Rivlin model was used to model the behavior of the IVD (Mustafy et al., 2014). The experimental values were used as the corresponding material constants (Casaroli et al., 2017).

The ligaments of the human spine used in the FE model included the anterior longitudinal ligament (ALL), posterior longitudinal ligament (PLL), ligamentum flavum (LF), intertransverse ligament (ITL), interspinous ligament (ISL), and supraspinous ligament (SSL). Their properties are listed in Table 2. The capsular ligament (CL) in the facet joint and connects the upper and lower vertebrae. This joint was modeled with a non-separate contact condition to mimic the behavior of the actual facet joint, which allows only limited slip and rotation while maintaining the contact spacing. Given that the ligament has characteristics identical to that of the spring, a tension-only characteristic was applied to the two-node beam element. As summarized in Table 2, each ligament exhibited a different strain based on small and large deformations (Goel et al., 1993; Chen et al., 2001; Chuang et al., 2013; Kim et al., 2015). The mesh generation, contact conditions, and loading conditions of the FE model were established using ANSYS workbench software (Version 2019 R1, ANSYS Inc., Pittsburgh, PA, United States).

### FE model of anteverted pelvis, NP, retroverted pelvis model

**FIGURE 4:**
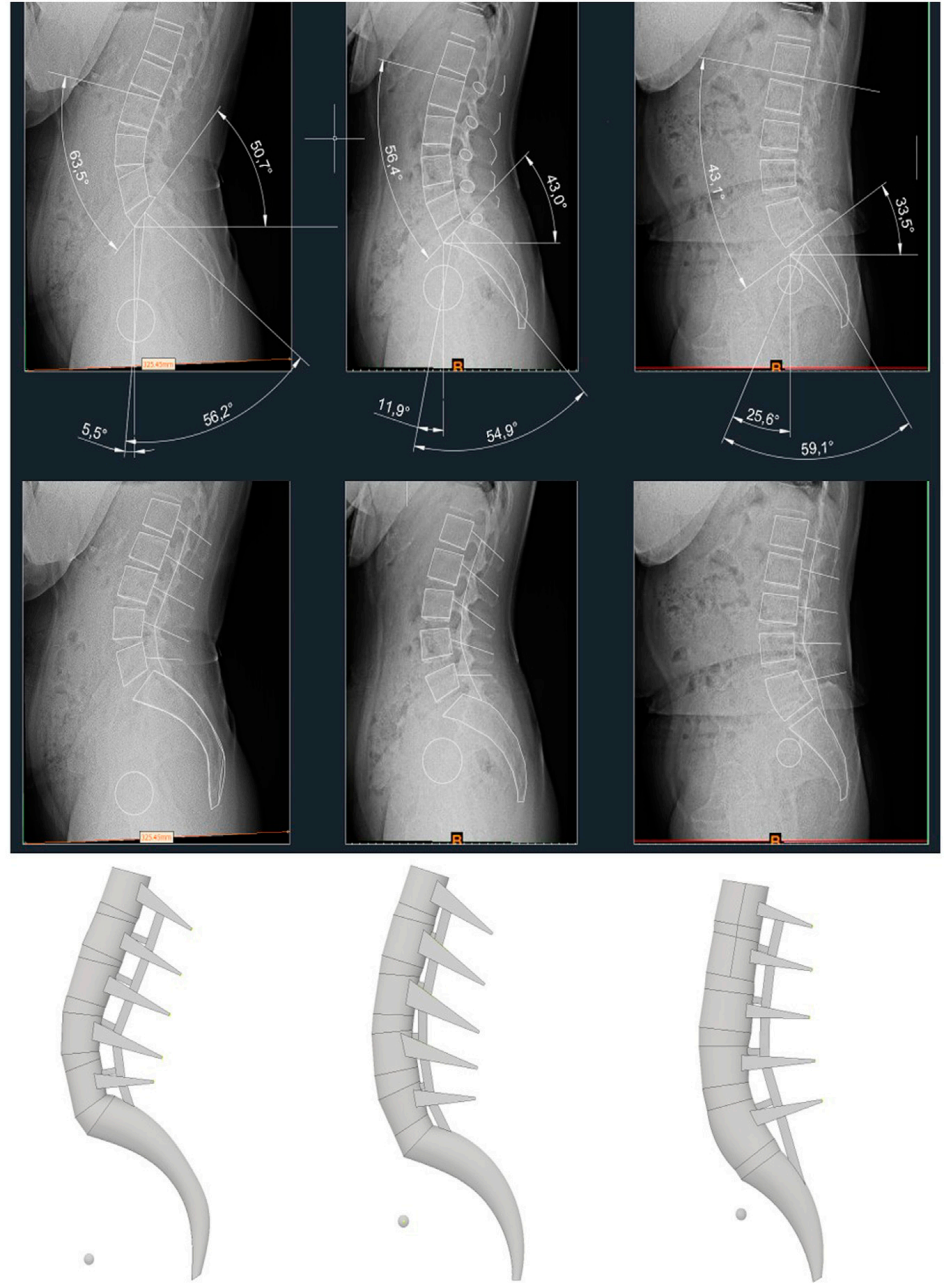
X-ray images displaying pelvic indicators of each representative model, and 3D models created based on it.

The radiologic protocol used in this study was standardized for all patients. The subjects were instructed to look straight ahead and stand in a comfortable position with their hips and knees fully extended and free of external support. Patients with a PT < 9° and an SS/PI > 0.80 were categorized into the anteverted pelvis (AP) group, and those with a PT > 17° and an SS/PI < 0.65 were categorized into the retroverted pelvis (RP) type (Choi et al., 2020). Subsequently, representatives were selected from each type. Each pelvic index identified by medical imaging is summarized in Table 1. A representative FE model was created using the process shown in Figure 4 for each pelvic type classified in this way.

## Model validation method

### Element optimization

**FIGURE 5:**
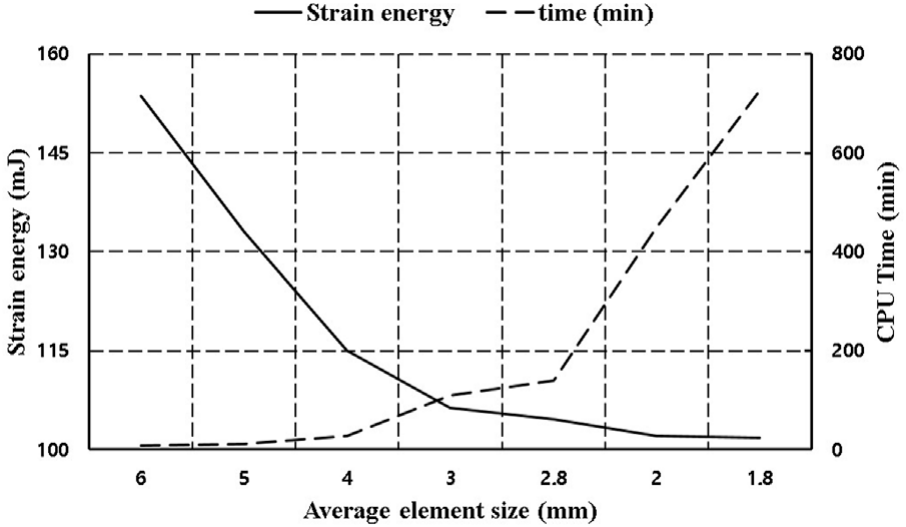
Analysis time and strain energy corresponding to element size.

Prior to model validation, element optimization analysis was performed by element size in the NP-type non-fusion model, and a pure moment of 7.5 Nm was applied to the upper surface of L1. Figure 5 shows the analysis time according to the total element size and total strain energy applied to the entire FE model for flexion under the condition of pure moment. The analysis CPU time rapidly for element sizes above 2.8 mm, and the strain energy converged to 100 mJ as the element size decreased. The optimum element size was determined to be 3 mm considering the appropriate analysis time and the precision of the solution in which the strain energy converged.

#### Loading condition

**FIGURE 6:**
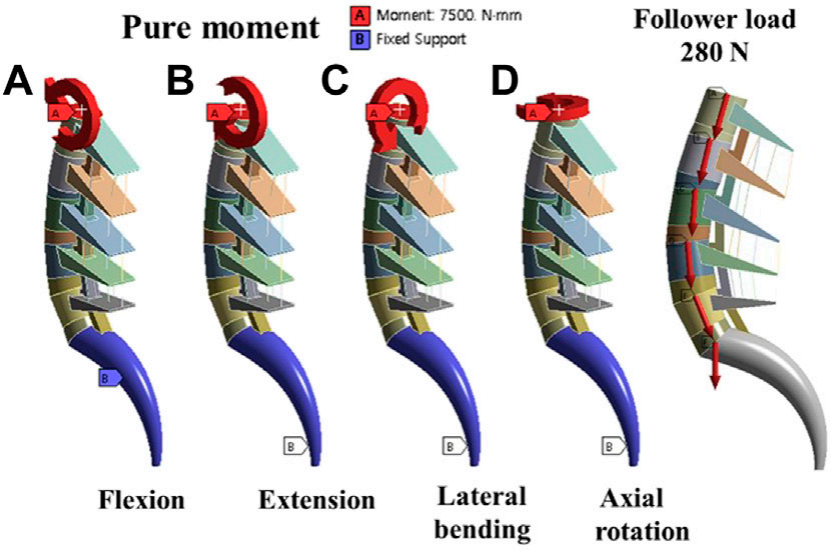
Boundary conditions; **(A)** Flexion motion **(B)** Extension motion **(C)** Lateral bending motion **(D)** Axial rotation motion and Follower load force.

The pure moment load condition for model validation was based on an *in vitro* study, in which the maximum possible load was applied without causing spinal damage to the multilevel lumbar spine. All degrees of freedom of the lower surface of S1 of the FE model were restricted to support the load, and a pure moment of 7.5 Nm was applied for all motions (Zhong et al., 2009), as shown in Figure 6.

The follower load of 280 N corresponded to the partial body weight of a person, and the moment of 7.5 Nm simulated the movement occurred in different conditions shown in Figure 6 (flexion, extension, bending, and axial rotation). Considering the symmetry of the sagittal plane, this study simulated the biomechanics of the fusion surgeries under four conditions: flexion, extension, bending-left, and rotation-left. The ROM, Fixation and cage stress, Peri-implant bone stress and Upper endplate of L5 were analyzed and exported.

#### Fusion model

The pedicle screw used for spinal fixation was 6.5 mm in diameter, the length of the spindle rod was designed to fit the length of each fixed segment, and the Φ6 titanium rod commonly used for spinal surgery was applied to reflect material properties (Jindal et al., 2012; Kim et al., 2021). The vertebrae and pedicle screws were held in a bonded condition, and were assumed to be completely immobilized. The cage was modeled based on the outer size of the INNESIS PEEK cage (BK Meditech Inc., Korea). The outer dimensions of the cage were 10 mm in height, 23 mm in length, and 11 mm in width. Three cage angles were used:0°, 4°, and 8°, and the material was made of PEEK (Kurtz and Devine, 2007).

**FIGURE 7:**
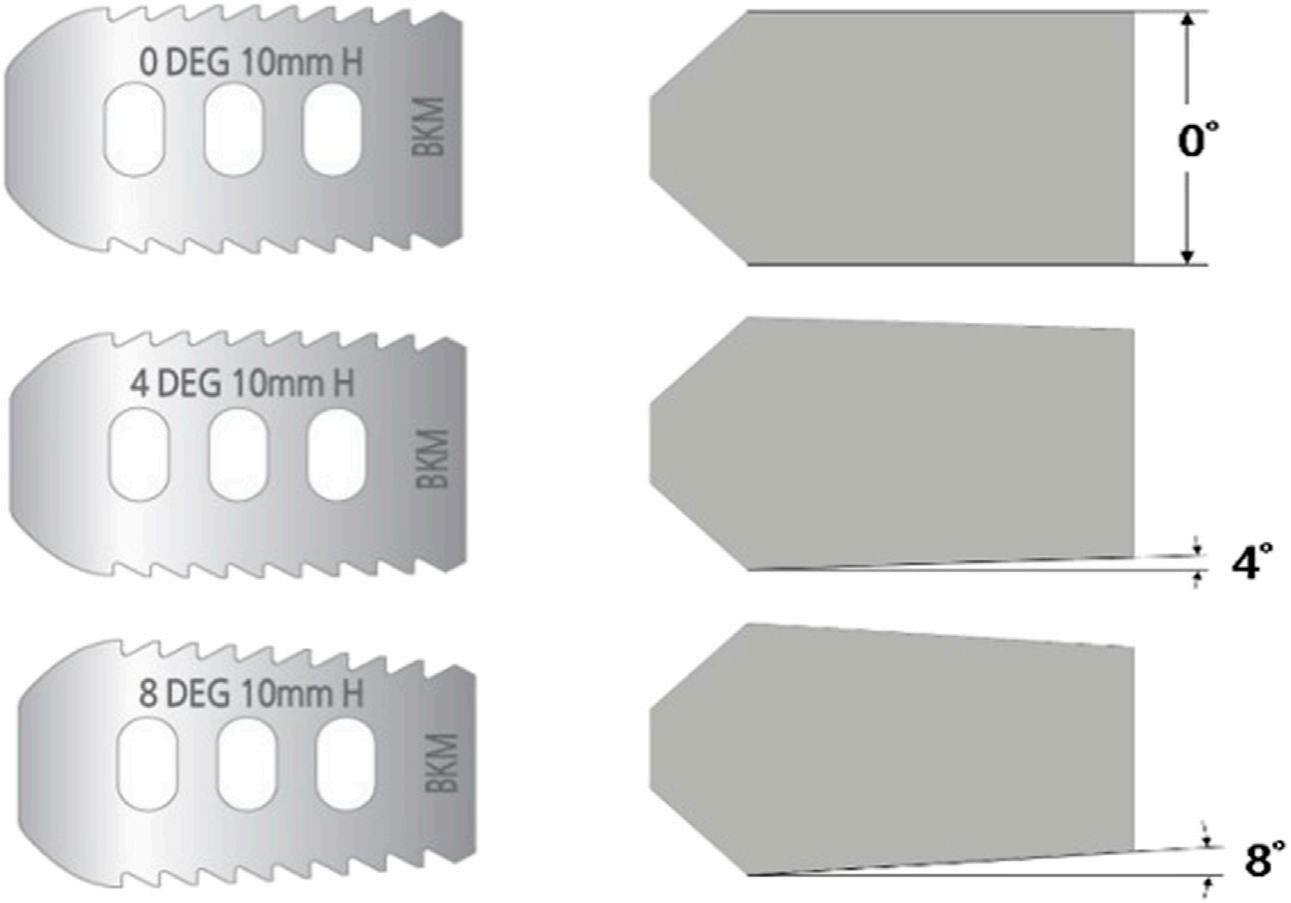
Simplified modeling of cage.

The cage shapes for each angle are shown in Figure 7, which shows shapes of the NP, AP, and RP types from the left and cage angles of 0°, 4°, and 8°, respectively. The cage was restrained in a bonded condition under the assumption that it was completely placed in the vertebrae without cage subsidence immediately after surgery.

## Results

### Model validation

To verify the FE model used in this study, the *in vitro* test results and ROM results of previous lumbar FE models were compared with those of the proposed model. However, only the NP-type ROM was compared because the *in vitro* test and other FEM studies also focused only on the general pelvic shape. Owing to the lack of *in vitro* experimental data or FEM study results based on the same spinal shape or sagittal parameters as those of the AP and RP types, the AP and RP types of ROMs were excluded from the comparison graph.

**FIGURE 8:**
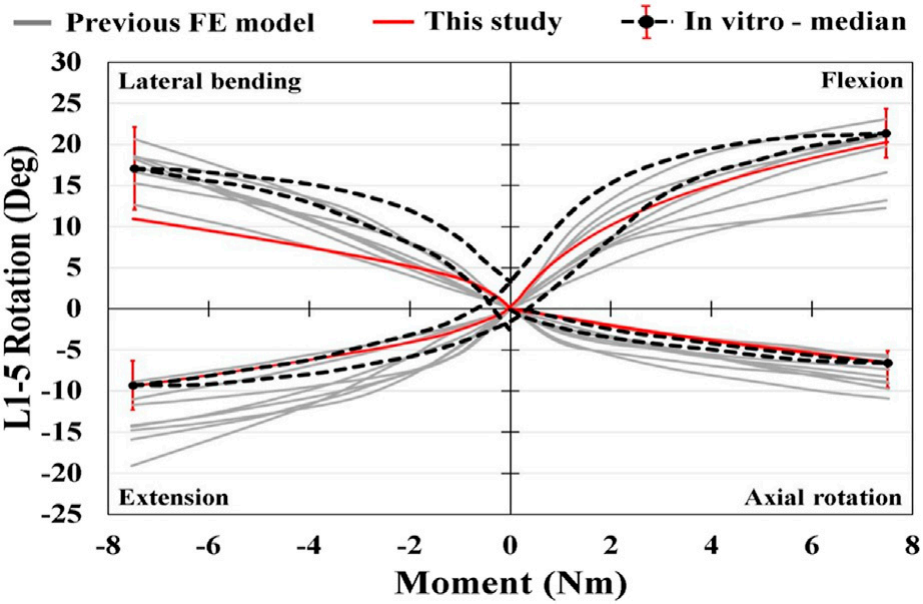
NP type of ROM for each motion.

Figure 8 shows a graph comparing the range of motion of the simplified model of this study under pure moment conditions of flexion, extension, lateral bending, and axial rotation, the cadaver experiment results, and the other FEM study results (Rohlmann et al., 2009; Dreischarf et al., 2014). The gray lines represent the range of the results of eight different FEM studies based on 3D medical images, and the dotted line represents the range of the cadaver experiment results. In the case of flexion, extension, and axial rotation, the results were similar to those of cadaver experiments compared to previous studies. Lateral bending was at the lower limit of the range in other FEM studies, but the difference was not significant.

### ROM according to pelvic type and cage angle

**FIGURE 9:**
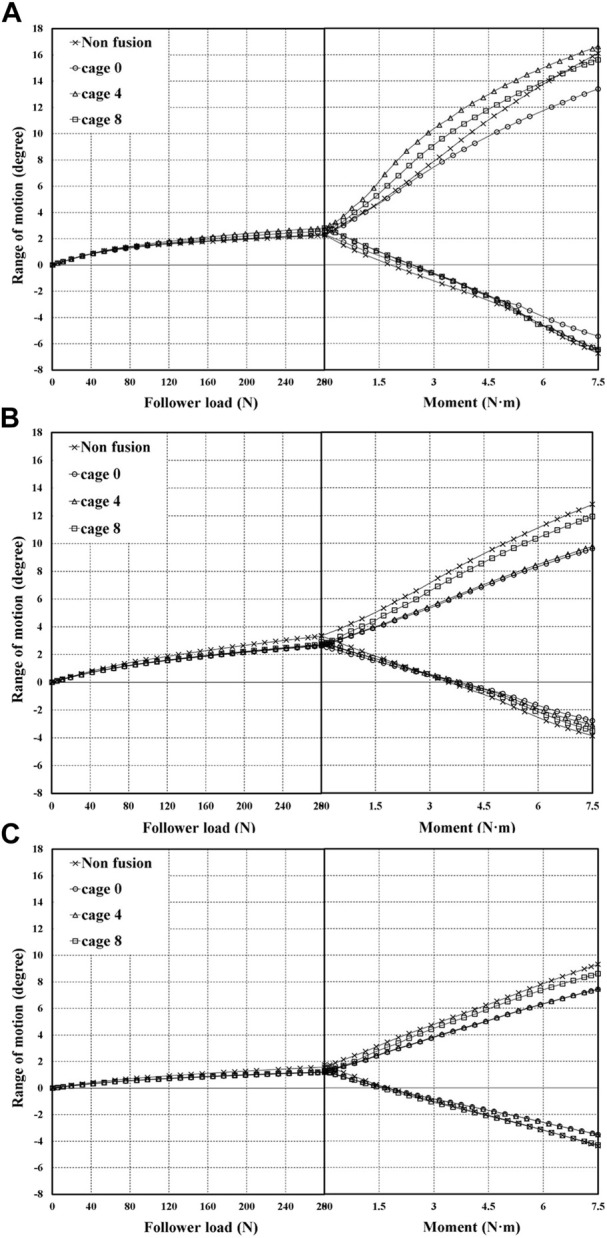
ROM generated by combination load **(A)** at NP type **(B)** at AP type **(C)** at RP type.

The ROM graph for extension under a combination load appears as shown in Figure 9 owing to compression by the follower load of Step 1 and tension by the moment of Step 2. In step 1, where only the follower load is applied, NP has the highest ROM in non-fusion and cage 0; in cage 4 and cage 8, AP exhibits a similar ROM level to NP. After the moment was applied in step 2, non-fusion exhibited a higher ROM than other fixation types, as expected, and the cage-inserted spine exhibited a similar ROM tendency. However, in the NP type, the ROM of cage 4 was higher than that of non-fusion.

As shown in Figure 9A, the case with the highest ROM for a combination load was the NP type implanted with cage 4. According to pelvic type, the NP type showed a relatively higher ROM than AP and RP. In the case of cage 0 implantation, the NP-type had a marginally lower ROM compared with the other cases, of which RP had the lowest ROM. Generally, the RP type shows a tendency toward low ROM even in step 1 with the follower load.

The average LL angles were 52.4°, 56°, and 39.7 °for the NP, AP, and RP types, respectively, as shown in Table 3. The difference between flexion and extension was expressed as the LF, and the results were analyzed. The NP type had the highest LF, followed by the AP type. Finally, RP type had the lowest LF. However, the AP type had the highest LL. In the pure moment, the average LF of the NP type was 24.47°, that of the AP type was 16.97°, and that of the RP type was 14.37°. For the combination load, the average LF for the NP type AP type, and RP type was 22.05°, 14.5°, and 11.8°, respectively. Furthermore, the average combination load applied by the follower force was 2.46° lower than that applied by the pure moment.

**TABLE 3:** Lumbar lordosis in non-fusion and flexion and extension at each cage angle.

Pure moment												
	Non-fusion (degree)			Cage 0 (degree)			Cage 4 (degree)			Cage 8 (degree)		
	Natural	Flexion	Extension	Natural	Flexion	Extension	Natural	Flexion	Extension	Natural	Flexion	Extension
NP	56.4	39.1	67.8	47.1	33.6	53.9	51.2	35.3	59.8	55.2	39.4	63.8
AP	63.5	51.1	70.2	49.7	39.2	54.9	53.5	44.0	58.7	57.3	44.7	63.1
RP	43.1	30.9	47.6	34.4	26.6	39.3	38.9	30.1	43.3	42.7	33.2	48.1
Combination load												
	Non-fusion (degree)			Cage 0 (degree)			Cage 4 (degree)			Cage 8 (degree)		
	Natural	Flexion	Extension	Natural	Flexion	Extension	Natural	Flexion	Extension	Natural	Flexion	Extension
NP	56.4	43.2	66.2	47.1	44.5	45.9	51.2	35.1	58.1	55.2	39.5	61.8
AP	63.5	51.1	67.8	49.7	39.8	52.7	53.5	43.7	57.0	57.3	45.9	61.0
RP	43.1	32.5	45.9	34.4	27.5	38.1	38.9	31.5	42.2	42.7	34.3	47.1

### Screw-spinal rod

**FIGURE 10:**
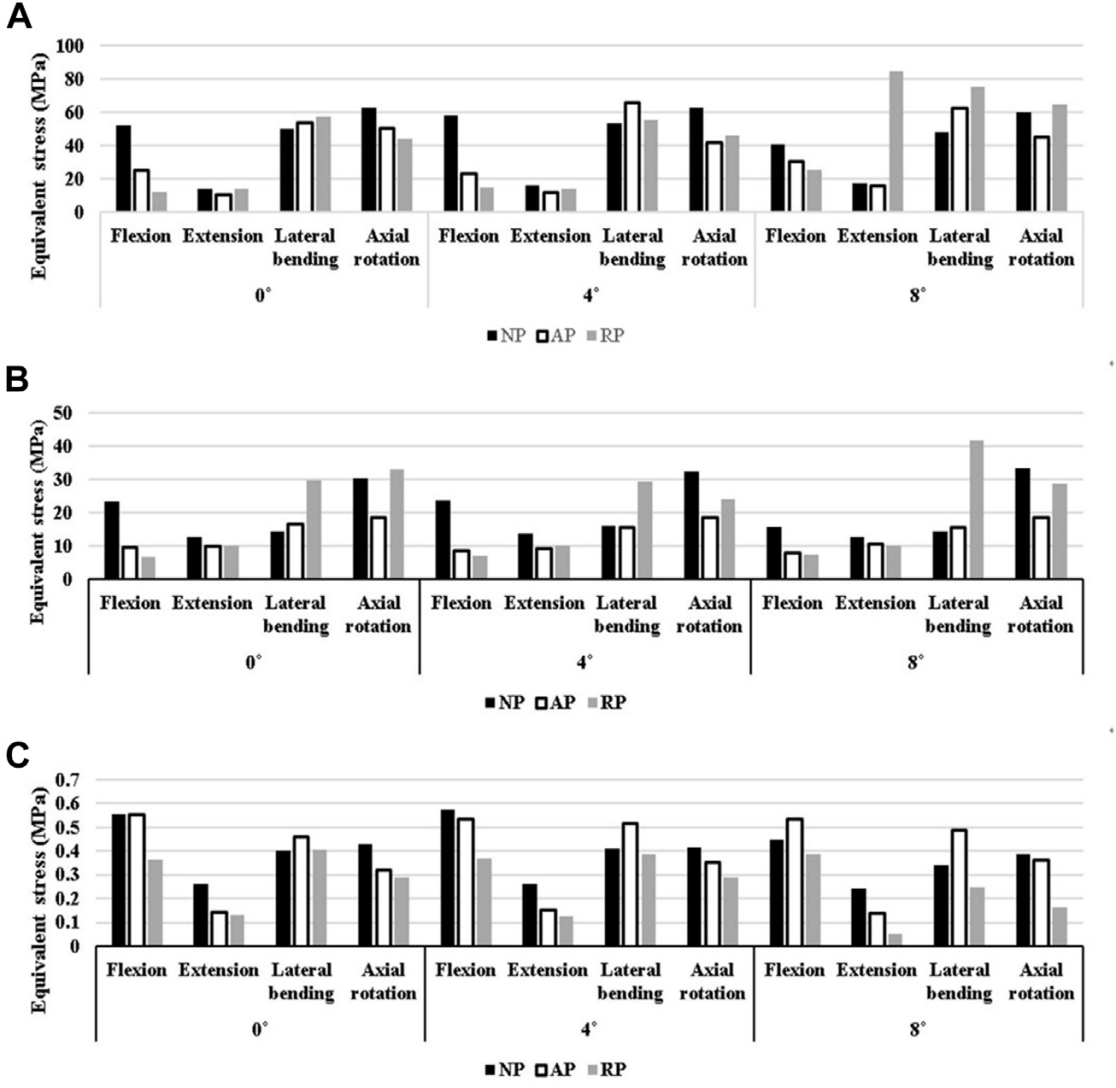
N Equivalent stress at each part. **(A)** at Screw-Spinal rod **(B)** at peri-implant **(C)** at L5 upper endplate.

In general, spinal fixation devices rarely break, however, screw failures occur because of repetitive motion and complex loads (Katonis et al., 2003). Figure 10A shows that the lowest equivalent stress applied to the screw and rod in the NP type is 41.4 MPa, which is 12.9% lower than that of cage 0, which has the highest equivalent stress. In the AP type, the equivalent stress is the highest for cage 0; the lowest stress, for cage 8, is 9% lower. In the case of the RP type, cage 0, which has a stress 48.8% lower than that of cage 8, had the smallest equivalent stress acting on the fixture. In cage 0, the equivalent stress of the RP type screw-rod was the lowest. For cage 0 and cage 4, the RP type, and for cage 8, the AP type generated a stress that was 28.5%, 31.4%, and 38.5% lower than the maximum stress, respectively.

### Peri-implant bone

The cause of fixation failure is breakage of the pedicle screw insertion site, rather than breakage of the fixation device itself (de Kunder et al., 2018). Therefore, the stability of the spinal fixation in each case was compared by calculating the equivalent stress around the vertebrae into which the fixation device was inserted in Figure 10B.

In the NP type, the case with the lowest stress in the peri-implant bone was cage 8, which was 11.4% lower than that in cage4, which exhibited the highest stress. In the AP type, cage 4 showed a 5.2% lower stress than cage 0, which had the highest stress, and in the RP type, cage 4 exhibited a 19.4% reduction in stress compared with cage8. In this case, the stress on the vertebra in contact with the screw was the lowest among all cases. In terms of the pelvic type, the AP type exhibits low stress in both cage 4 and cage 8 as well as in cage 0. Compared to the highest stress results, the levels were reduced by 32.4%, 39.8%, and 40.1%, respectively.

### L5 upper endplate

The direction of the gravity load causes the cage to apply pressure to the endplate of the L5 upper. Therefore, stress shielding can occur at this time (Chang et al., 2020). The NP type generated a stress 14.3% lower than the highest stress, which was generated in cage 8; the AP type stress was 5% lower when cage 0 was used compared to the case where the upper part of L5 had the highest stress, as shown in Figure 10C. For the RP type, cage 8 exhibited a stress that was 28.7% lower than the maximum equivalent stress. Comparing only the magnitude of the average equivalent stress generated by the cage on the upper surface of L5 regardless of the pelvic group, the RP group exhibited a relatively lower average equivalent stress than the other groups at all cage angles.

## Discussion

Clinicians use flexion and extension motions in routine clinical examinations to evaluate lumbar flexibility. However, the exact effect of the lumbar and pelvic shapes on anterior and posterior flexion during sagittal movement is unclear. Salem (Salem et al., 2018) reported that the correction/restoration of sagittal balance has been inconsistently reported and has varied from modest or insignificant at the levels instrumented (Uribe et al., 2012) to substantial corrections of up to 20° (Knight et al., 2009). Intraoperatively, surgeons typically rely on a cross-table lateral radiograph to determine the sagittal alignment of the spinal segment in question. However, the amount of correction retained following surgery remains undetermined.

Due to the above study results, it was determined that it was difficult to numerically analyze the degree of sagittal balance restoration after LIF in clinical studies. Accordingly, in this study, LIFs were simulated using virtual surgery through the pelvic shape to which the patient’s pelvic index was applied. And as a result, the equivalent stress and sagittal parameters of each region of the lumbar spine were calculated. If the sagittal plane pelvic index according to the cage angle and pelvic shape is analyzed by a numerical analysis method, the results can be numerically confirmed. Therefore, if the data of this study are accumulated and standardized, it is considered to be an indicator that can assist actual surgery.

**FIGURE 11:**
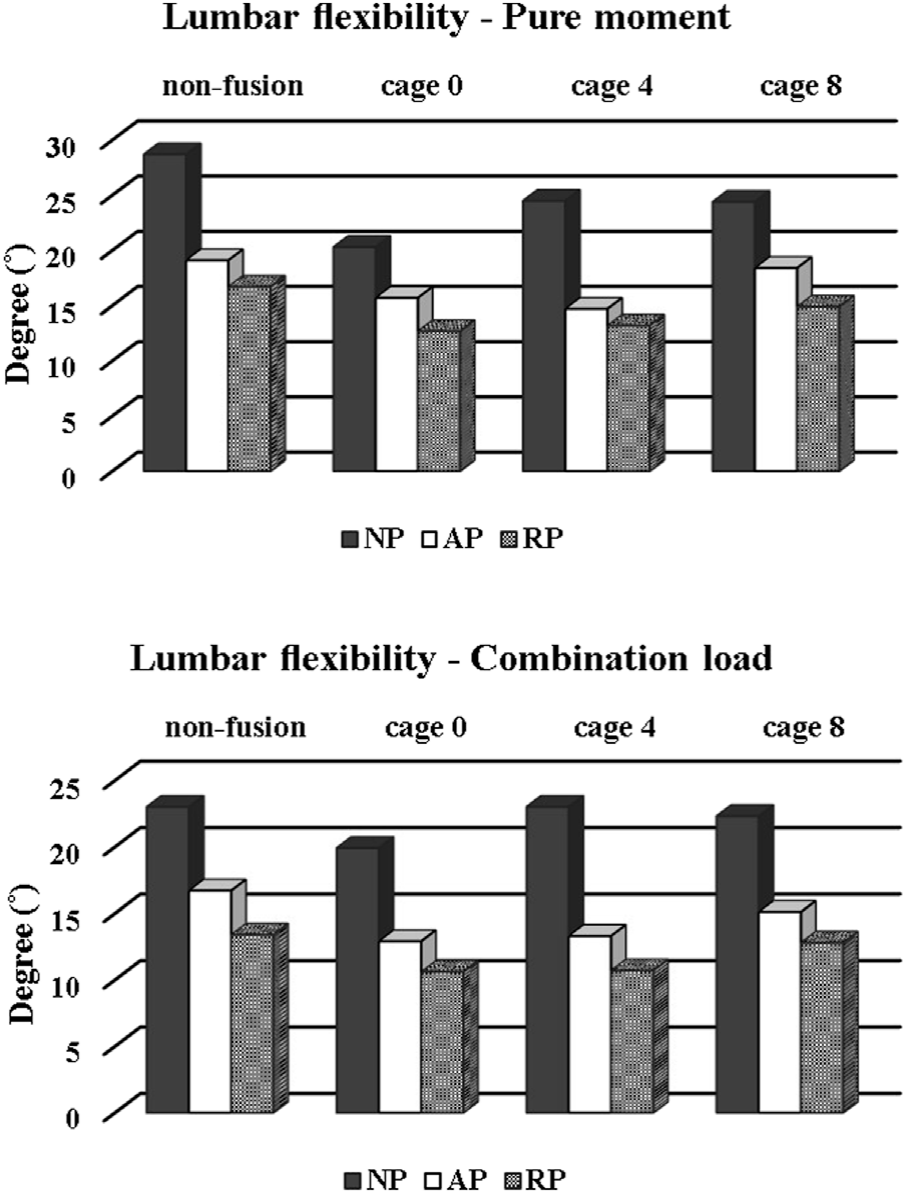
Lumbar flexibility by cage type and pelvis type.

Accordingly, in this study, pelvic shapes were classified into NP, AP, and RP types based on sagittal parameters, and flexion and extension were performed for each pelvic shape using finite element analysis. And load controlled methods (LCM) were used for spinal motion. According to Chuang’s study, the displacement controlled method (DCM) model has high calculation efficiency (Chuang et al., 2013), but produces a relatively high equivalent stress result compared to the load controlled methods (LCM) or ROM controlled methods (RCM) model. In other words, using DCM has the potential to lead to higher-than-actual stress results. It has also been reported that the DCM should be used cautiously for the kinematic and mechanical investigation of the caudal region. In addition, the RCM model has higher reliability of mechanical stress and kinematic results compared to the physiological model, but it takes a long time to calculate and thus the efficiency is lowered. Zhong reported that DCM is suitable for evaluation of the patient’s daily life motion during restoration after surgery and LCM is suitable for evaluating the patient’s normal life work-loading condition after surgery (Zhong et al., 2009). Therefore, simulation was performed by adopting an LCM suitable for the purpose of this study with calculate efficiency and reliability. Figure 11 shows that LF according to the motion of each pelvis shape was analyzed by comparing the LL in the neutral, maximum flexion, and maximum extension states according to pelvic shape.

LL in the neutral state was highest for the AP type with an average of 56°, less for the NP type with 52.47°, and lowest for the RP type with 39.77°. However, LF was the highest for NP type at 24.47, lower for AP type at 16.97, and lowest for RP type at 14.37 under the condition of pure moment. If LL and LF were directly related, the AP type would possess the largest LF as well as LL. However, the observed result is exactly the opposite of what was expected. However, as the RP type exhibits small values of LL and LF, LF is attributed to various factors rather than a variable that is completely dependent on LL (Cappuccino et al., 2010; Zhang et al., 2018). Additionally, the difference in ROM according to cage angle was confirmed in the combination load. The joint is anatomically weak. Thus, the pedicle screw is susceptible to damage if a large moment is applied (Park et al., 2021). This study analyzed the equivalent stress of the screw rod by motion according to the pelvis shape and cage angle.

Even in the fixed state of the same segment, the stress applied to the screw and vertebrae differed marginally, depending on the pelvis shape and cage angle. In the case of the screw, the average equivalent stress acting on cage 8 was the highest depending on the cage angle, regardless of the pelvis type; cages 0 and 4 were subjected to similar stresses. The pelvic type with the lowest stress was cage 0 of the RP type. For cage angles according to the different pelvis types, the lowest stress occurred in cage 8 for NP and cage 0 for AP. Conversely, when cage 0 for the NP type, cage 8 for the AP type, and cage 8 for the RP type were used, the stress increased by 14.9%, 9.9%, and 95.3%, respectively, compared to the case with the lowest stress. When cage 4 was used, the posterior and peri-implant bones showed the lowest level of equivalent stress after spinal correction in all pelvic types. In contrast, when cage 0 was used for the NP type, it exhibited a high equivalent stress compared with the other fixed states. Therefore, when cage 0 is used for the NP type, the clinician also needs to consider the simulation results.

Zhang reported that the maximum stress in the cage decreased significantly with an increase in the angle of lordosis (Zhang et al., 2018); in contrast, the maximum stress of the endplate increased as the angle of lordosis increased. In this study, the RP type, which had a relatively low LL, exhibited the lowest level of stress on the endplate at the top of the cage. The NP and AP types had the highest equivalent stress when cage4 was used, and the RP type had the highest stress at cage0. However, the difference between the stresses was insignificant. Consequently, significant difference in stress was observed between the cage angles. In addition, because the stress value applied to the L5 top endplate was not large, the possibility of subsidence owing to stress shielding in general motion was confirmed to be extremely low.

However, this study has several limitations, and further studies should be implemented to obtain highly accurate predictive models. First, the actual human body has various pelvic shapes. Thus, the results of the proposed model cannot be expected to represent all cases, considering that the simulation was performed on a limited model after selecting the representative pelvis type. Therefore, in future studies, a greater number of representative model samples of the same pelvis type must be used. In addition, the sagittal plane trend must be analyzed more closely according to the pelvic shape through clinical results and FEM studies for each group. Another limitation of this study is that it ignored the deformation amount and equivalent stress of the cage, which was modeled as simply as possible. Furthermore, the actual vertebral body has a slight curve on the inside. However, in this study, the surface of the vertebral body was created as a simplified model and implemented as a flat surface. This simplified model was used for the ease of calculating the contact surface between the cage and body part of the vertebrae. However, owing to the simplicity of the model, the angle change of the LL after cage insertion may differ from the actual one.

Finally, only static analysis was performed in this study, in which only the follower load generated by the patient’s own weight and the moment due to motion was considered. In future studies, various external and repetitive loads that occur when walking or running must be considered to ensure that the simulations reflect the actual operating conditions of the pelvic system.

## Conclusion

Among the pelvic types in this study, the NP type exhibited the highest ROM and LF. The comparison of the NP and RP types indicates that LL affects ROM and lumbar flexibility. However, the relationship between the AP and RP types does not corroborate this dependence of ROM and FL on LL.

Furthermore, ROM was more affected by pelvic type than by cage angle. However, cage angle of 0° exhibited limited ROM regardless of the pelvic type. In addition, the difference in the equivalent stress between the fixation device and vertebra due to the cage angle was extremely small.

## Data Availability

The original contributions presented in the study are included in the article/Supplementary Material, further inquiries can be directed to the corresponding author.
